# Testing Dose-Dependent Effects of the Nectar Alkaloid Anabasine on Trypanosome Parasite Loads in Adult Bumble Bees

**DOI:** 10.1371/journal.pone.0142496

**Published:** 2015-11-06

**Authors:** Winston E. Anthony, Evan C. Palmer-Young, Anne S. Leonard, Rebecca E. Irwin, Lynn S. Adler

**Affiliations:** 1 Department of Biology, University of Massachusetts Amherst, Amherst, Massachusetts, United States of America; 2 Department of Biological Sciences, Dartmouth College, Hanover, New Hampshire, United States of America; 3 Department of Applied Ecology, North Carolina State University, Raleigh, North Carolina, United States of America; San Diego, UNITED STATES

## Abstract

The impact of consuming biologically active compounds is often dose-dependent, where small quantities can be medicinal while larger doses are toxic. The consumption of plant secondary compounds can be toxic to herbivores in large doses, but can also improve survival in parasitized herbivores. In addition, recent studies have found that consuming nectar secondary compounds may decrease parasite loads in pollinators. However, the effect of compound dose on bee survival and parasite loads has not been assessed. To determine how secondary compound consumption affects survival and pathogen load in *Bombus impatiens*, we manipulated the presence of a common gut parasite, *Crithidia bombi*, and dietary concentration of anabasine, a nectar alkaloid produced by *Nicotiana* spp. using four concentrations naturally observed in floral nectar. We hypothesized that increased consumption of secondary compounds at concentrations found in nature would decrease survival of uninfected bees, but improve survival and ameliorate parasite loads in infected bees. We found medicinal effects of anabasine in infected bees; the high-anabasine diet decreased parasite loads and increased the probability of clearing the infection entirely. However, survival time was not affected by any level of anabasine concentration, or by interactive effects of anabasine concentration and infection. *Crithidia* infection reduced survival time by more than two days, but this effect was not significant. Our results support a medicinal role for anabasine at the highest concentration; moreover, we found no evidence for a survival-related cost of anabasine consumption across the concentration range found in nectar. Our results suggest that consuming anabasine at the higher levels of the natural range could reduce or clear pathogen loads without incurring costs for healthy bees.

## Introduction

Plant secondary compounds play an essential role in mediating plant-herbivore interactions [1]. Concentrations of plant secondary compounds can vary widely both among plant species [2–5] as well as among individuals within a species [6]. This variation can strongly affect plant resistance to herbivores as well as herbivore performance [4, 7]. Secondary compounds are not limited to vegetative tissue, but can also be found in flowers and floral rewards, including nectar [8, 9]. Like leaf chemistry, nectar secondary compound concentrations also vary among individuals within species [10] as well as across species [2, 5, 11]. For example, caffeine concentrations varied over an order of magnitude in nectar of different *Coffea* species, reaching levels as high as instant coffee in *C*. *canephora* [11]. Pollinators can show strong dose-dependent responses to nectar secondary compounds (reviewed in [12]). For example, low levels of nectar caffeine in artificial flowers increased pollen deposition by bumble bees relative to control flowers without caffeine. However, higher caffeine levels were deterrent, resulting in pollen deposition equivalent to control flowers [12]. Similar studies have found that low levels of secondary compounds can be attractive to honey bees, while higher levels are deterrent [13, 14]. Thus, assessing effects of secondary compounds at multiple concentrations is critical to understand their role in ecological interactions.

Secondary compounds generally reduce herbivore survivorship and reproduction, but can also benefit herbivores by reducing parasitism or predation ([15, 16], reviewed in [17, 18]). Compound dose can affect herbivore parasitism, but the direction of this effect depends on the compound, the herbivore and the parasite [19]. In pollinators, recent studies have demonstrated that a range of nectar secondary compounds can reduce *Crithidia bombi* loads or slow the progression of infection in bumble bees [20–22]. In honey bees, thymol consumption improved longevity of bees infected with *Nosema ceranae* [23], and consumption of sunflower nectar reduced *N*. *ceranae* loads [24]. However, there can be costs as well as benefits associated with consuming secondary compounds. Chronic consumption of nicotine had detrimental effects on healthy but not infected *Bombus terrestris* [22]. Similarly, consumption of the nectar alkaloids gelsemine and anabasine reduced *C*. *bombi* loads in *B*. *impatiens* but also decreased measures of reproduction [20, 21]. In spite of the wide variation in secondary compound concentration in plant tissues, including nectar, previous studies manipulated the presence rather than concentration of secondary compounds. To date, we have little information on the associated costs and benefits of varying concentrations of nectar compounds for parasite load and bee performance. Assessing the relationship between compound dose, parasite loads, and host mortality could define concentrations at which compounds ameliorate infection severity without compromising survival.

The goal of this study was to assess whether the nectar alkaloid anabasine exerts dose-dependent effects on survival and parasite load in bumble bees infected with *C*. *bombi*. In *Nicotiana glauca*, the mean nectar anabasine level in one study was 5.0 ppm [25], while a subsequent study by the same research group found mean nectar concentrations less than 0.5 ppm [26], indicating substantial variation across plants and years. We predicted that consuming high concentrations of dietary anabasine would decrease survival time in healthy bees. In infected bees, however, we expected that costs of anabasine consumption would be offset by anti-parasite benefits, such that increasing concentrations of anabasine would decrease parasite loads and improve survival. Wild bees provide essential pollination services [27], but many bumble bee populations are dwindling [28]. A variety of potential factors are implicated in declines [29], including the parasites *C*. *bombi* and *N*. *bombi* [30]. Assessing the role of natural variation in nectar secondary compound concentrations on parasite loads and bee survival may shed new light on strategies for managing bee disease.

## Materials and Methods

### Study system

Our experiments were conducted on laboratory colonies of *B*. *impatiens* (Apidae), the Common Eastern Bumble Bee, a eusocial insect distributed throughout the eastern US [28]. Queens winter underground, emerging in spring to found nests. The colony grows throughout the summer, during which workers collect nectar and pollen from local flowers. In autumn, colony production shifts from workers towards males (drones) and new queens [31]. As generalist foragers, *B*. *impatiens* likely encounter an array of nectar and pollen secondary compounds in their diet [32].

*Crithidia* is a genus of arthropod-parasitizing trypanosome protozoa that are related to the causative agents of leishmaniasis, Chagas disease, and African sleeping sickness. *Crithidia bombi* prevalence can reach as high as 80% of bees infected in North American and European *Bombus* [30, 33–35]. *Crithidia bombi* resides in the hindgut and is transmitted vertically by overwintering queens and horizontally through bumble bee feces in nest material [36] and at flowers [37]. The ability of *C*. *bombi* to infect multiple *Bombus* species gives it the potential to play a large role in bumble bee decline [38]. *Crithidia bombi* infection can increase hibernation mortality in overwintering queens, decrease queen fitness by 40%, and reduce colony size and production of males [39]. *C*. *bombi* resistance is compromised in colonies with low genetic diversity [40, 41], making this parasite especially detrimental to small inbred populations. *Crithidia bombi* infection can also inhibit bee learning and foraging efficiency, thereby impacting pollination services [42, 43].

Anabasine is a pyridine and piperidine alkaloid found in plants of the *Nicotiana* genus. A structural isomer of nicotine, anabasine caused dose-dependent growth inhibition of a variety of bacterial and fungal pathogens of humans [44]. In humans, low levels of anabasine provoke nausea, and vomiting; high levels cause paralysis and death [45]. In insects, anabasine was toxic to mosquito larvae [46] and apple aphids [47]. In Palestinian sunbirds (*Nectarinia osea*), anabasine in *N*. *glauca* nectar deterred feeding, reduced sugar assimilation, and decreased gut transit time [48]. Anabasine at 5 ppm reduced *C*. *bombi* loads in *B*. *impatiens* by 81%, and 20 ppm reduced parasite loads but also delayed time to egg laying [21]. However, this study did not consider the effects of multiple anabasine concentrations.

### Experimental Design

To determine how anabasine consumption and *C*. *bombi* infection interact to affect bumble bee survival, we fed bees with four concentrations of anabasine: 0 ppm, 1.81 ppm, 3.62 ppm, and 7.25 ppm (hereafter referred to as control, low, medium and high). These concentrations were chosen to span the range of anabasine found in *N*. *glauca* nectar; the highest concentration was approximately 2 SD above the mean in a previous study [48]. The four anabasine treatments were crossed in a factorial design with two infection treatments (*C*. *bombi* infected or control), for a total of eight treatment combinations. Bees from six uninfected colonies of *B*. *impatiens* (Biobest, Leamington, ON, Canada) were randomly assigned to treatments as they emerged from puparia. The experiment was partitioned into 20 blocks of eight for a total of 160 bees. A block was defined as a group of eight consecutively emerging bees that included all treatment combinations. After excluding bees that died fewer than three days after inoculation, one colony that contributed only 3 bees to the experiment, or those without final weight measurements, there was a total of 131 bees included in analysis.

Colonies were housed in plastic acrylic hives contained within 30 cm x 20 cm x 20 cm cardboard boxes. They were fed every 2 days with a 5 g portion of pollen (Koppert Biological Systems, Scarborough, Ontario, Canada) mixed with 30% sucrose solution and given *ad libitum* access to 30% sucrose solution. Pupal clumps containing callow workers about to emerge were removed from colonies twice weekly. To remove pupal clumps, workers were evacuated from the colony using an Insect-Vac (BioQuip, Rancho Dominguez, CA, USA) and clumps were removed with forceps and scalpels. Pupal clumps were kept at 26.5°C with a 12-hour photoperiod in a separate ventilated 500 mL plastic container (Rubbermaid, Fairlawn, OH, USA) for each colony. Pupal clumps were monitored twice daily for newly emerged workers (callows). By preventing contact of experimental bees with their natal colonies, we avoided exposing them to microorganisms within the colony that can affect growth and spread of *C*. *bombi* [49]. Newly emerged bees were randomly assigned to experimental treatments and individually housed in 25 ml plastic scintillation vials (see S1 Fig) from the time they emerged until dissection. Bees were maintained on a laboratory bench at room temperature with artificial light during the day and natural light from nearby windows.

### *Crithidia bombi* inoculation and experimental treatments

*Crithidia bombi* was obtained from gut dissections of wild *B*. *impatiens* collected at the University of Massachusetts (Amherst, MA).The parasite was propagated in two laboratory "source" colonies of *B*. *impatiens* that were inoculated with *C*. *bombi* from the wild bees. Colonies were infected by placing five 200 μl droplets of sucrose containing 15,000 parasite cells μL^-1^ in the colonies' honeypots, allowing multiple bees to be inoculated by consuming the droplets. In addition, for each inoculation approximately 10 bees were pulled from the hive and individually inoculated with a 6 μL droplets containing 6,000 *C*. *bombi* cells. We monitored the spread of infection for approximately four weeks in the source colonies by sampling feces from haphazardly chosen workers [50]. Colonies were considered ready for use as source colonies when at least 5 out of 10 haphazardly sampled worker bees exhibited infection levels exceeding 800 *C*. *bombi* cells μL^-1^ of gut homogenate.

To infect experimental worker bees, inoculum was prepared by dissecting 5 bees from an infected source colony. Mid- and hindguts were ground with a plastic pestle in 300 μL of distilled water in a 2 mL microcentrifuge tube. The solution was vortexed for 5 seconds and allowed to settle for 3–8 hours at room temperature. Parasite cells from a 10 μL aliquot of the supernatant were counted with a haemocytometer under a compound microscope at 40x magnification. Gut extracts were combined with sucrose and distilled water to achieve a solution that was 30% sucrose (wt/wt) with 1,500 *C*. *bombi* cells μL^-1^. Freshly prepared inoculum was used for each day of experimental inoculations.

The experiment was conducted during June and July, 2012, following the protocol used to test effects of gelsemine and other secondary compounds on *C*. *bombi* [20, 51]. For the first 2 days after emergence, bees were fed a 30% sucrose solution *ad libitum*. At the end of the second day, the feeder was removed and the bee was starved overnight. On the morning of the third day, we inoculated experimental bees with 6,000 *C*. *bombi* cells in 4 μL 30% sucrose; uninfected bees received 4 μL 30% sucrose without *C*. *bombi*. After inoculation, bees were fed 0.5 ml of their treatment solution (30% sucrose with no, low, medium or high anabasine) and approximately 20 mg of pollen daily.

### Survival and parasite loads

Following inoculation, we checked bees daily and recorded mortality. Bee mass (at time of death) and length of the radial cell on the right forewing were recorded as estimates of bee size [52]. We then dissected dead bees to assess parasite load. The gut was removed from the abdomen with forceps and *C*. *bombi* cells were counted using the protocol under ‘*C*. *bombi* inoculation.’ Two uninoculated bees that contained *C*. *bombi*, presumably through mislabeling or contamination during treatments, were excluded from analyses.

### Statistical analysis

Pathogen responses were analyzed with SAS Version 9.4 (Cary, NC) and survival analyses were conducted using R Version 3.2.2 [53]. For inoculated bees only (60 bees total, with n = 13–16 per treatment group), the effect of anabasine treatment on *C*. *bombi* parasitism was analyzed two ways. First, we used type III SS mixed-model ANOVA to analyze the effect of anabasine treatment as a fixed effect on parasite loads (cell concentration mL^-1^ gut extract, log[x+1] transformed); colony and block were included in the model as random effects. Counts were log(x+1) transformed to improve residual fit to a normal distribution and retain samples with zero counts. Second, we used logistic regression to determine whether anabasine treatment, colony, or block affected the probability of having detectable infection at time of death. Neither colony nor block were significant in this model and were removed. We used the SAS ESTIMATE statement to calculate post-hoc significance tests comparing odds of infection between treatment levels. For inoculated and uninoculated bees (131 bees total; with n = 12 to 21 per treatment combination), effects of diet and infection on survival were analyzed using a Cox proportional hazards mixed-effects model [54]. Death hazard rate was used as the response variable; anabasine treatment, infection, and their interaction were used as predictor variables; colony and block were included as random factors. Statistical significance of main effects and the interaction term were tested using Wald χ^2^ tests [55]. For the analyses of survival and *C*. *bombi* cell counts, radial wing cell length and callow mass were initially included as covariates, but were then excluded because neither explained significant variation in either response.

## Results

**Fig 1:**
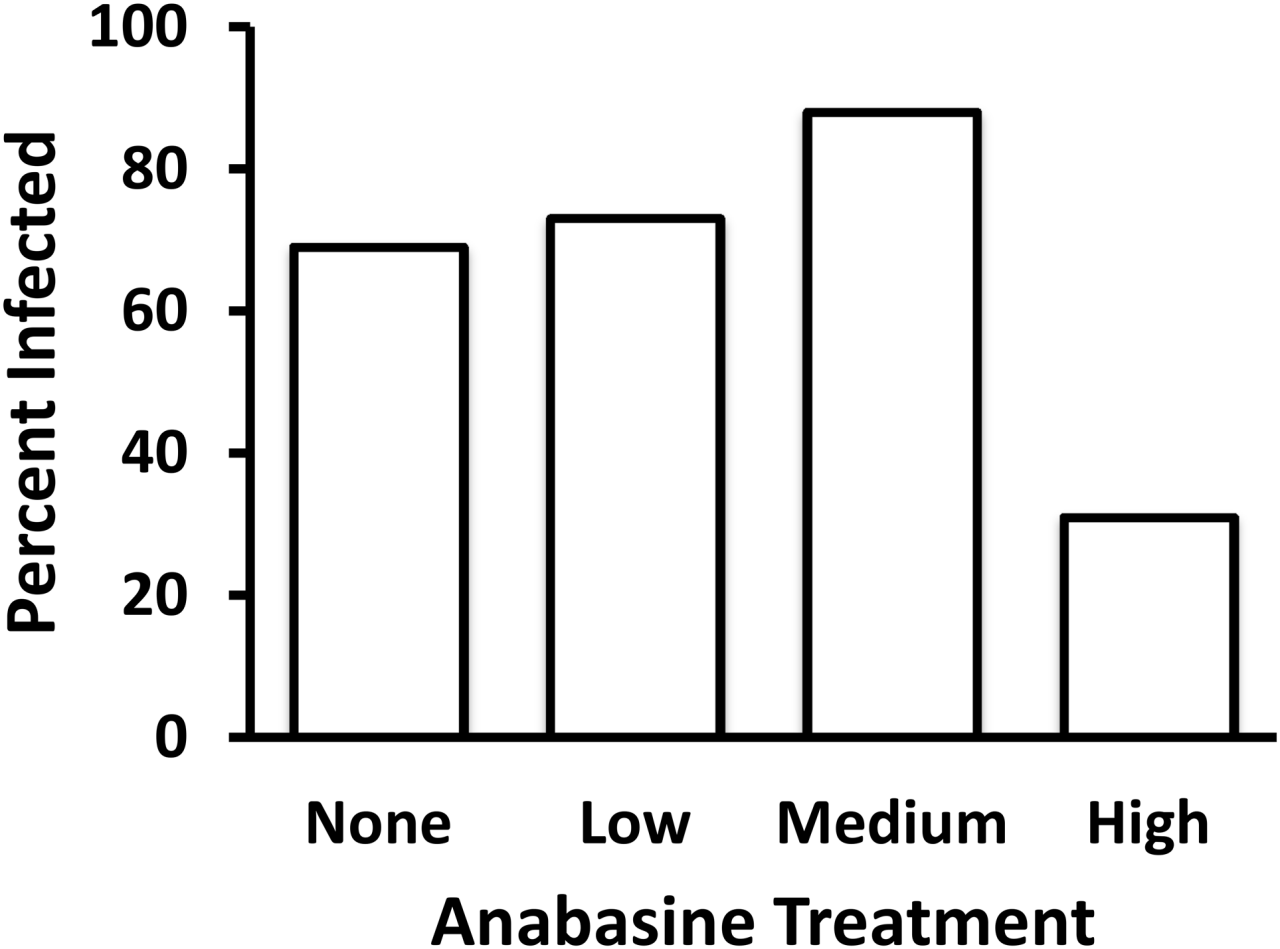
Effects of anabasine treatment on probability of infection at time of death. Anabasine treatment significantly affected the mean probability of bees having detectable *C*. *bombi* at time of death (P = 0.0256).

**Fig 2:**
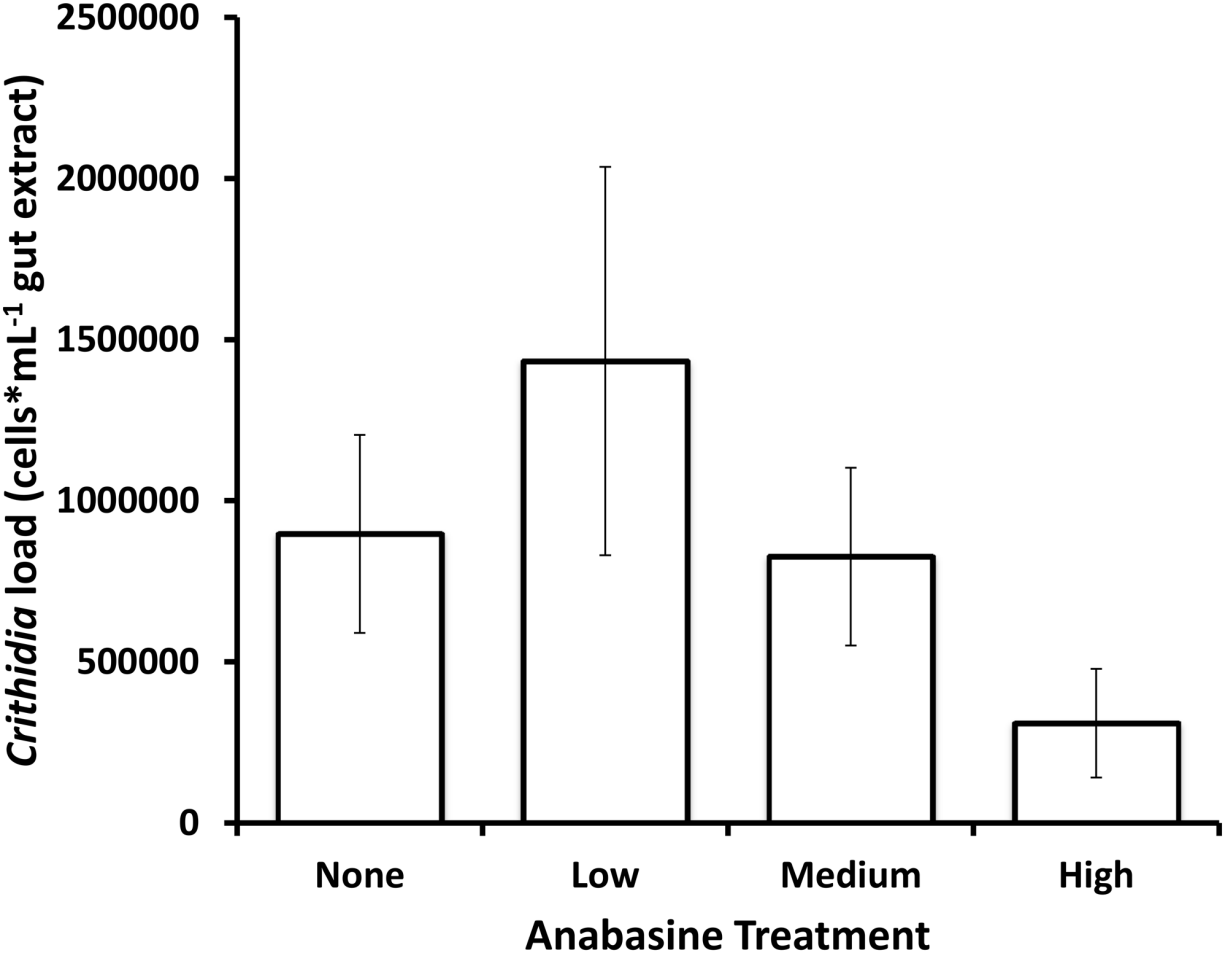
Effects of anabasine treatment on parasite load. Anabasine treatment had a significant effect on *C*. *bombi* parasite load at time of death (P = 0.015). Error bars show mean ± SEM of untransformed data.

There was a significant effect of the anabasine treatment on the probability that bees had detectable *C*. *bombi* at death (df = 3, Wald Chi-Square = 9.2925, P = 0.0256). Post-hoc tests found near-significant differences between the medium and high-anabasine treatment (z = 1.96; P = 0.0503) but not other comparisons of individual treatments (|z| < 1.3, P > 0.2). However, the high-anabasine treatment was significantly different from all other treatments combined (z = -2.93; P = 0.0034); final infection rate in the high-anabasine group was less than half of that in any other treatment group (Fig 1). Anabasine treatment also affected mean parasite load (F_3,56_ = 3.79, P = 0.015), again with the lowest *C*. *bombi* cell counts in the high anabasine treatment. Pairwise comparisons with a Tukey’s test showed a significant reduction in parasite loads in the high anabasine treatment compared to the medium treatment (P = 0.0126, Fig 2) and marginally different between the high and the low treatment (P = 0.051). None of the other pairwise comparisons were significantly different (P > 0.1 for all).

Survival was not significantly affected by anabasine treatment (χ^2^ = 1.4, df = 3, P = 0.71), *Crithidia* infection (χ^2^ = 0.47, df = 1, P = 0.49), or their interaction (χ^2^ = 0.32, df = 3, P = 0.96).

## Discussion

Within the concentration range observed in floral nectar, anabasine had no significant effects on bumble bee survival but improved resistance to the trypanosome parasite *C*. *bombi*. Interestingly, anabasine only reduced pathogen loads at the highest concentration, suggesting that there is a threshold effect rather than linear relationship with dose (Figs 1 and 2). Thus, we did not find the hypothesized dose-dependent relationship, with medicinal benefits at low doses and toxic effects at high doses. Instead, there was little cost or benefit of anabasine consumption at low doses, and the only medicinal effect was realized at the highest dose. Our result is unlikely to be due to effects of starvation on pathogens when nectar alkaloid concentrations are high, as prior work found that *B*. *impatiens* consumed 16% more sucrose solution with 20 ppm anabasine than an anabasine-free control [21]. This study contributes to a growing body of literature that examines the effects of secondary compounds on pollinator health. Further research, including field study, is needed to determine the effects of anabasine and other secondary compounds on wild populations subjected to multiple stressors and exposed to mixtures of secondary compounds. Knowledge of the antiparasitic properties of secondary compounds could eventually be applied to bee management.

We had predicted that anabasine ingestion would decrease longevity of healthy bees, but improve survival in infected bees. To our surprise, neither *C*. *bombi* infection, anabasine concentration, nor their interaction significantly affected survival. The absence of survival-related costs of alkaloid consumption indicates that bees have high tolerance for this alkaloid under laboratory conditions. Our results are consistent with previous experiments showing high deterrence thresholds and no effects on mortality for bees consuming nicotine—an isomer of anabasine—at levels far exceeding those found in nectar [56]. Anabasine consumption might be more costly under the energetic stresses found under field conditions, particularly because nectar phytochemicals and nicotine analogues can decrease total food consumption [56, 57]. Similarly, *C*. *bombi* infection may have more detrimental effects on starved bees [58], suggesting that bees in natural conditions might be more susceptible to alkaloid effects than sedentary bees fed *ad libitum*. Alternatively, because our experimental bees were maintained individually on a lab bench, exposed to light and variable temperatures, they may have been more stressed than colony bees and died due to other causes before experiencing mortality effects of alkaloid consumption. In support of this hypothesis, a related experiment using *B*. *terrestris* inoculated with *C*. *bombi* found that mortality effects of nicotine consumption were only apparent 30–35 days after treatments began [22], while our bees died on average after 16 days.

Anabasine consumption reduced bumble bee parasite loads at the highest concentration tested, which was still in the range of natural nectar values [25]. This result is consistent with previous studies that documented reduced parasitism in bees fed alkaloids [59], including nicotine [60] and anabasine, as well as other types of nectar secondary metabolites [23, 51]. Most of this prior work tested compounds over relatively short time frames. Our study builds on these prior findings by showing that reduced pathogen loads are sustained over the course of a worker bee’s lifespan, and that high anabasine increased the chances of clearing the infection entirely. This is in contrast to recent work in *B*. *terrestris*, where consuming nicotine reduced *C*. *bombi* loads initially but did not clear infections, and within 10 days pathogen levels in nicotine-fed bees approached that of control bees [22]. In that study, chronic nicotine consumption was detrimental to healthy but not infected bees, supporting the hypothesis that such consumption can be medicinal. Given that bees voluntarily consume secondary compounds at levels exceeding those in nectar [56], and that infected bees may prefer nectar with secondary compounds over control solutions, the medicinal effects of anabasine have the potential to be realized under field conditions.

Dietary context may enhance or diminish the medicinal effects of consuming individual compounds. The dietary concentrations used in our experiments would be approached by bees consuming exclusively *N*. *glauca* nectar, which is possible given that worker bees tend to forage on one or several floral species [31]. But medicinal effects might be realized at lower concentrations if compounds have synergistic effects when consumed as mixtures. Even single-plant diets include multiple secondary chemicals; *N*. *glauca* nectar, for example, contains both nicotine and anabasine [48]. Diets consumed by less specialized foragers would include a variety of metabolites, as would the diets of nest bees consuming the pollen and honey derived from different host plants.

The medicinal effects of particular dietary components may depend on a three-way interaction between parasite genotype, host genotype, and diet composition. In *B*. *terrestris*, the effects of food supplementation on *C*. *bombi* loads depended on host genotype-parasite genotype interactions, such that the ideal sugar concentration differed by bee colony and parasite strain [61]. Bumble bee gut microbes, which differ between colonies, were protective against *C*. *bombi* infection [62]. The microbiome, which is known to influence drug metabolism in humans [63], might determine how colonies metabolize and respond to secondary compounds. Genotypic variation among parasites in susceptibility to secondary compounds may also alter the course of infection. Our experiment used six different colonies, but tested only one parasite strain. Future work should address local and regional variability in parasite tolerance to medicinal metabolites.

Anabasine consumption reduced mean pathogen loads and increased the odds of clearing infections, but we did not examine changes in within-hive or population-level transmission resulting from alkaloid consumption. However, recent work suggests that these individual-level benefits may be realized at the scale of the colony as well; nectar anabasine reduced *C*. *bombi* levels in microcolonies by 65% [51]. The consequences of anabasine consumption for population-level transmission dynamics are unknown. Microcolonies of *B*. *impatiens* fed 20 ppm anabasine consumed significantly more liquids than bees fed control solutions, indicating that anabasine also increases rates of excretion in bees [51]. Although gut and fecal *C*. *bombi* concentrations are highly correlated [50], suggesting that anabasine could lower the infectiousness of workers by decreasing parasite loads, alkaloid-induced gut flushing might actually promote the spread of parasite cells between colonies by increasing fecal contamination of nests and flowers. This hypothesis could be tested by comparing parasite transmission at flowers visited by bees that consume anabasine-containing versus control diets.

The effects of secondary compounds on bumble bee infection should be considered in an applied context. The identification of anti-parasitic compounds will suggest possible supplements for managed bees; thymol has already been shown to reduce *N*. *ceranae* [23] and *Varroa jacobsoni* [64] loads in honeybee colonies. Parasites might rapidly evolve resistance to individual compounds, however. A more holistic approach would be the provision of multiple medicinal compounds, possibly in the form of deliberately planted metabolite- and species-rich floral arrays. Studies assessing the efficacy of different means of supplementation against diverse parasite assemblages are of potential interest to the fields of ecology, wildlife management, and agriculture.

Our study demonstrated that consuming natural levels of nectar anabasine can reduce lifetime parasite loads and increase the likelihood of clearing infection entirely. Continued unraveling of the relationship between bee diet, parasite resistance, and pollination services would be of agricultural, ecological, and evolutionary interest. Future laboratory and field research can answer basic questions about host-parasite-plant evolution and ecology while identifying practical solutions to bee decline.

## Supporting Information

S1 Fig Diagram of bee vial and feeder. Bees were housed individually in scintillation vials with *ad libitum* access to treatment solution. A 1.5 mL microcentrifuge tube was inserted into a 10 mm diameter hole in the vial's cap. The microcentrifuge tube was filled with the appropriate anabasine solution and plugged with a 10 mm long, 10 mm diameter wick of dental cotton. The vial also had four 1 mm diameter holes in the side for ventilation.
(PPTX)

S1 Data Data used for statistical analyses. The first worksheet contains data used for the analysis of survival time. The second worksheet contains data used for analysis of probability of infection and parasite load. The third worksheet describes the variable names in the first two worksheets.
(XLSX)
