# Supplementary figures and images for: Testing Dose-Dependent Effects of the Nectar Alkaloid Anabasine on Trypanosome Parasite Loads in Adult Bumble Bees
Source: PLoS One. 2015 Nov 6;10(11):e0142496. doi: 10.1371/journal.pone.0142496 (PMC4636389; doi:10.1371/journal.pone.0142496)

## Slide 1
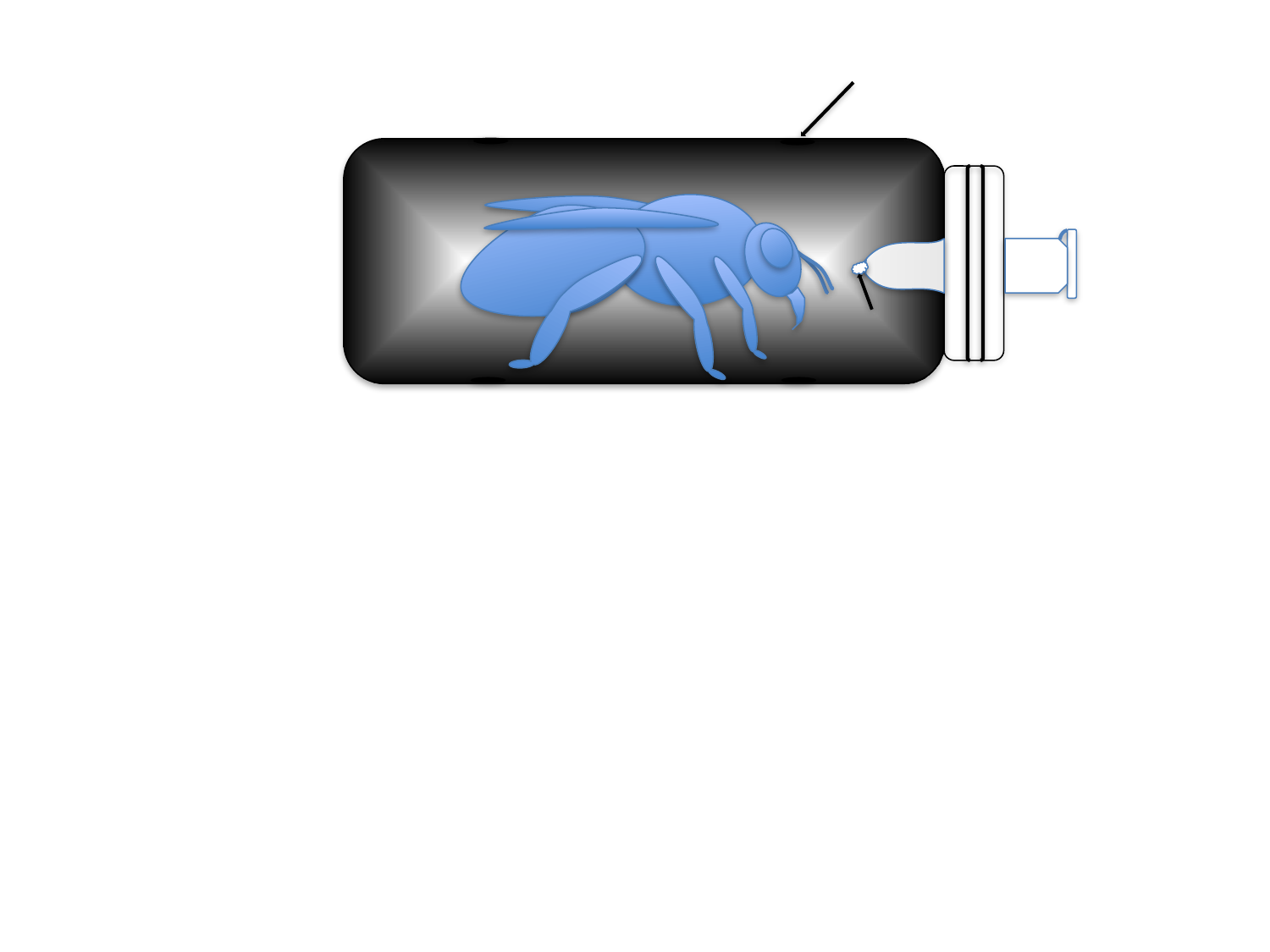

Supplement: S1 Fig — Bees were housed individually in scintillation vials with ad libitum access to treatment solution. A 1.5 mL microcentrifuge tube was inserted into a 10 mm diameter hole in the vial's cap. The microcentrifuge tube was filled with the appropriate anabasine solution and plugged with a 10 mm long, 10 mm diameter wick of dental cotton. The vial also had four 1 mm diameter holes in the side for ventilation. (PPTX) [file pone.0142496.s001.pptx]
